# Supplementary material for: An open-source framework for large-scale, flexible evaluation of biomedical text mining systems
Source: J Biomed Discov Collab. 2008 Jan 29;3:1. doi: 10.1186/1747-5333-3-1 (PMC2276192; doi:10.1186/1747-5333-3-1)
Supplement: Additional file 1 — Evaluation platform source code. The additional file contains the software evaluation framework discussed in this paper. The most current release can be downloaded from SourceForge.net [1]. See the accompanying README file for instructions on installation and use. Also included in the distribution is a collection of UIMA wrappers for some commonly used BioNLP tools and annotated corpora. [file 1747-5333-3-1-S1.zip › bionlp_uima_repository-1.0.1/docs/api/edu/uchsc/ccp/util/nlp/parser/corpus/Bio1CorpusParser.html]

Bio1CorpusParser (Center for Computational Pharmacology Util/UIMA API)


|  |  |  |
| --- | --- | --- |
| **Overview** \| **Package** \| **Class** \| **Tree** \| **Deprecated** \| **Index** \| **Help** | |  |
| PREV CLASS   **NEXT CLASS** | **FRAMES**    **NO FRAMES**     **All Classes** |  |
| SUMMARY: NESTED \| FIELD \| CONSTR \| METHOD | DETAIL: FIELD \| CONSTR \| METHOD |  |


---


## edu.uchsc.ccp.util.nlp.parser.corpus Class Bio1CorpusParser

```
java.lang.Object
  edu.uchsc.ccp.util.nlp.parser.corpus.CorpusParser
      edu.uchsc.ccp.util.nlp.parser.corpus.Bio1CorpusParser
```

**All Implemented Interfaces:**: Iterator

---

``` public class Bio1CorpusParser extends CorpusParser ```

A parser for the Bio1 corpus. This class outputs the original text and accompanying annotations for each Bio1 corpus document. Titles and abstracts
are output as separate documents.   
  
Output Classes:  

- protein
- DNA
- RNA
- cell line
- cell type
- virus
- tissue
- organism
- organism type 2
- subcellular location

**Author:**
:   williamb

---

| **Field Summary** | |
| --- | --- |
| `static int` | `DOCUMENT_COLLECTION_ID` |


**Constructor Summary**

`Bio1CorpusParser(String bio1CorpusFile)`


| **Method Summary** | |
| --- | --- |
| `void` | `close()` |
| `static void` | `determineOverlapWithGENIA(String bio1CorpusFile, String geniaCorpusXMLFile)`             The documents in the Bio1 corpus overlap somewhat with those in the GENIA corpus. |
| `boolean` | `hasNext()` |
| `static void` | `main(String[] args)`             To determine overlap in documents with GENIA:  args[0] = parse or compare; if parse, the Bio1 corpus is parsed. |
| `GenericDocument` | `next()` |
| `static void` | `parseBio1Corpus(BufferedReader br)`             This method parses the corpus in one pass... |

**Methods inherited from class edu.uchsc.ccp.util.nlp.parser.corpus.CorpusParser**

`createTextAnnotation, error, remove, warn`

**Methods inherited from class java.lang.Object**

`clone, equals, finalize, getClass, hashCode, notify, notifyAll, toString, wait, wait, wait`

**Field Detail**

### DOCUMENT\_COLLECTION\_ID

```
public static final int DOCUMENT_COLLECTION_ID
```

**See Also:**: Constant Field Values


**Constructor Detail**

### Bio1CorpusParser

```
public Bio1CorpusParser(String bio1CorpusFile)
                 throws IOException
```

**Throws:**: `IOException`


**Method Detail**

### hasNext

```
public boolean hasNext()
```

:   **Specified by:**: `hasNext` in interface `Iterator` **Specified by:**: `hasNext` in class `CorpusParser`

---


### next

```
public GenericDocument next()
```

:   **Specified by:**: `next` in interface `Iterator` **Specified by:**: `next` in class `CorpusParser`

---


### parseBio1Corpus

```
public static void parseBio1Corpus(BufferedReader br)
                            throws IOException
```

:   This method parses the corpus in one pass... useful when all annotations are needed at one time, for instance when inserting them into a
    database. This method was created prior to implementing the Iterator interface, thus some code duplication exists. TODO: Rewrite method to
    utilize Iterator methods, thereby removing duplicate code

    :   **Parameters:**: `br` - **Throws:**: `IOException`

---


### determineOverlapWithGENIA

```
public static void determineOverlapWithGENIA(String bio1CorpusFile,
                                             String geniaCorpusXMLFile)
                                      throws IOException
```

:   The documents in the Bio1 corpus overlap somewhat with those in the GENIA corpus. This method examines that overlap.

    :   **Parameters:**: `bio1CorpusFile` -: `geniaCorpusXMLFile` - **Throws:**: `IOException`

---


### close

```
public void close()
           throws IOException
```

:   **Specified by:**: `close` in class `CorpusParser`
:   **Throws:**: `IOException`

---


### main

```
public static final void main(String[] args)
```

:   To determine overlap in documents with GENIA:  
    args[0] = parse or compare; if parse, the Bio1 corpus is parsed. If compare, the overlap of ids with GENIA is computed  
    args[1] = bio1 corpus file   
    args[2] = genia xml corpus file  
    args[3] = connectionpropertiesfile

    :   **Parameters:**: `args` -


---


|  |  |  |
| --- | --- | --- |
| **Overview** \| **Package** \| **Class** \| **Tree** \| **Deprecated** \| **Index** \| **Help** | |  |
| PREV CLASS   **NEXT CLASS** | **FRAMES**    **NO FRAMES**     **All Classes** |  |
| SUMMARY: NESTED \| FIELD \| CONSTR \| METHOD | DETAIL: FIELD \| CONSTR \| METHOD |  |


---
